# Factors Influencing Intention to Leave Among Nurse Managers: A Cross‐Sectional Study

**DOI:** 10.1111/wvn.70135

**Published:** 2026-04-01

**Authors:** Valentina Simonetti, Ilaria Franconi, Natascia Piermattei, Andrea Faragalli, Marco Fioretti, Donatella Rossolini, Giulia Santamaria, Rosaria Gesuita, Giancarlo Cicolini

**Affiliations:** ^1^ Department of Innovative Technologies in Medicine & Dentistry “G. d'Annunzio” University of Chieti‐Pescara Chieti Italy; ^2^ Center of Innovation in Nursing Research (CINR) – Center for Advanced Studies and Technology (CAST) “G. d'Annunzio” University of Chieti‐Pescara Chieti Italy; ^3^ Operating Room Salesi Children's Hospital, AOU Delle Marche Ancona Italy; ^4^ AST Ancona Ancona Italy; ^5^ Centre of Epidemiology, Biostatistics & Medical Information Technology, Department of Biomedical Sciences and Public Health Marche Polytechnic University Ancona Italy; ^6^ AST Ascoli Piceno Ascoli Piceno Italy; ^7^ IRCCS INRCA Ancona Italy

## Abstract

**Aims:**

To estimate the proportion of Italian nurse managers (NMs) intending to leave (ITL) their positions and to identify associated socio‐demographic, job‐related, and psychosocial factors.

**Design:**

Cross‐sectional study.

**Methods:**

Between September and November 2023, 464 NMs from 19 public hospitals completed a case‐report form and the short version of the Copenhagen Psychosocial Questionnaire II (COPSOQ II). Latent Class Analysis (LCA) identified ITL profiles, and multiple logistic regression assessed factors associated with ITL.

**Results:**

284 NMs (61.2%; 95% CI 57–66) reported an intention to leave within 12 months. LCA identified two classes: (1) Low‐ITL (54%)—mainly outpatient NMs from Central regions with strong relationships with management, good support, work–life balance, and autonomy (55.9% probability of being unlikely to leave). (2) High‐ITL (46%)—mainly surgical or critical‐care NMs, often from Northern regions, marked by poor management relations, low support and high work–family conflict (80.9% probability of being likely to leave). Multiple regression confirmed that stronger management relations reduced ITL (OR 0.60, 95% CI 0.46–0.79) whereas high job demands and work–health conflict increased it (OR 1.56, 95% CI 1.19–2.04). Northern location also predicted higher ITL (OR 1.58, 95% CI 1.03–2.44). Demographics, education, and clinical setting were not significantly associated.

**Linking Evidence to Action:**

These findings suggest that healthcare organizations should prioritize managerial and organizational strategies targeting modifiable work‐related factors to reduce nurse managers' intention to leave. Interventions aimed at improving organizational support, work environment, and job satisfaction may contribute to workforce retention at the managerial level. Future research should evaluate the effectiveness of targeted organizational interventions in sustaining nurse manager retention.

## 1 Introduction

The nursing profession is facing a worldwide shortage of qualified staff (Both‐Nwabuwe et al. 2018) and the “retention” of nursing staff, defined as the planned commitment to build an environment that motivates employees to remain in their position (World Health Organization. Regional Office for Europe and South‐Eastern Europe Health Network 2011), has become a priority in health‐care organizations (Spence Laschinger et al. 2012). Nowadays, the phenomenon of the “intention to leave” defined as a worker's intention to leave his/her current position or organization (Cho et al. 2009) has a remarkable impact on the entire healthcare systems (Efendi et al. 2019), as it represents a significant indicator of staff turnover (Cheng and Liou 2011) negatively affecting patient outcomes and care costs (Liou 2009). Therefore, it is extremely urgent to identify and implement strategies that effectively reduce this phenomenon (Kantek and Kaya 2017). Both organizational and individual factors—such as emotional exhaustion, personal accomplishments, physical health, and psychological well‐being—significantly impact nurses' intention to leave (World Health Organization 2020). Importantly, burnout, job satisfaction and the work environment have been consistently identified in the literature as important factors influencing the decisions of nurses and nursing managers to leave their organization (Brown et al. 2013; Kantek and Kaya 2017; Labrague 2024).

Within work environments, nurse managers (NMs)—recognized as Top or Middle managers (Calamandrei and Orlandi 2015; Filomeno et al. 2023)—play a pivotal role in balancing the needs of their staff with those of patients and their families (Prestia 2020); they also contribute to mitigating nursing staff shortages (Han and Jekel 2011; Hill 2011), thereby positively influencing the quality of care provided (Aiken et al. 2011; Mackoff and Triolo 2008). Consequently, when NMs leave their positions, the provision of patient care may be compromised, potentially leading to an increase in the number of adverse events (Warshawsky et al. 2013). However, according to Warshawsky and Havens (2014), it is not reasonable to assume that the factors influencing NMs' intention to leave are identical to those affecting nursing staff, but further research is needed to explore this issue more comprehensively (Hewko et al. 2015; Warshawsky and Havens 2014). Current evidence indicates that burnout, job changes, retirement, and job promotion are the first four more frequently reported reasons for NMs to express their intention to leave (Warshawsky and Havens 2014).

In addition, a wide range of organizational factors (values and culture, opportunities for life‐long learning and professional development, adequacy of human and financial resources, administrative systems, leadership, effectiveness, and confidence in employers), together with role‐related factors (levels of support, degree of control, accountability and role expectations), as well as personal factors (recognition, family commitment, professional qualification, personality and temperament, emotional maturity and job satisfaction), all affect NMs' intention to leave (Brown et al. 2013; Laschinger et al. 2013).

Empowered nurse managers at all levels who feel supported by their healthcare organizations are more likely to remain in their position, stay committed to achieving quality patient care, and serve as influential role models for potential future leaders (Laschinger et al. 2013). Supportive workplace wellness cultures and a stronger sense of mattering have also been associated with lower burnout and fewer mental health problems among nurse managers (Melnyk et al. 2024).

For these reasons, it is essential to pursue a better working environment (Montgomery and Patrician 2022) taking into account workers' well‐being, which enhances job satisfaction (Laschinger et al. 2013), reduces stress (Lowe 2013), and burnout (Brown et al. 2013; Rushton et al. 2015), and strengthens nursing manager resilience in order to achieve positive results across organizational levels (Warshawsky et al. 2016).

Consistently, the “psychosocial work environment”, defined as a set of socio‐structural opportunities within the workplace that enable individuals to meet their expectations for well‐being, productivity, learning and positive social interactions (Siegrist and Marmot 2004), is considered a crucial element in shaping a healthy organizational climate (European Commission: Directorate‐General for Employment, S. A., and Inclusion 2000; Cox et al. 2000).

Multiple studies have reported factors influencing the retention of nursing staff (Park et al. 2019; Sasso et al. 2019). In contrast, little attention has been given to the retention, intent to stay or leave, or actual turnover of NMs (Laschinger et al. 2013; Penconek et al. 2024).

## 2 Aim

The primary aim of this study is to estimate the prevalence of job‐leaving intention among NMs and to identify the factors associated with these intentions, considering socio‐demographic variables, workplace characteristics, and psychosocial work environment conditions.

## 3 Methods

### 3.1 Research Design

A cross‐sectional multicenter study was conducted between September and November 2023.

### 3.2 Recruitment Criteria

NMs (top, upper‐middle, and lower‐middle managers) working in 21 public hospitals were invited to participate in the survey (Table S1).

NMs who did not consent to participate, did not sign the informed consent, those off duty for over 3 months, and those with temporary or assistance tasks, were excluded.

### 3.3 Data Collection, Variables, and Measures

A case report form was developed and structured into three sections:

- Socio‐demographic section: participants provided information on age, gender, number of children, marital status and education.
- Job section: this section collected information on professional experience (years of experience in the nursing profession), position experience (years in the current managerial role), work setting, and number of staff managed, geographical area of work setting. A multiple‐choice check‐box format item was included to identify motivations that may contribute to the intention to leave their job. In addition, participants were asked “How likely would you be to leave your current employment at your hospital within the next year?”, with responses expressed on a 4‐point Likert scale (Extremely likely, likely, Unlikely, Not at all likely).

Psychosocial work environment: The Italian version of the Copenhagen Psychosocial Questionnaire II (COPSOQ II)‐short version (Setti et al. 2017a) was administered to investigate the psychosocial working conditions. The COPSOQ II‐short version consists of 30 items, grouped in 16 scales, covering the following dimensions: quantitative demands, work pace, emotional demands, decision authority, skill discretion, predictability, rewards, role clarity, quality of leadership, supervisor's support, job satisfaction, work–family conflict, trust, justice, self‐rated health, burnout. Among these scales, 14 were made of two items, while the remaining two were single‐item. The items had four to five options, depending on the domain, evaluated through the Likert scale, with a score ranging from 0 (never/very little disagreement) to 3 or 4 (always/agreement). For comparability, the questionnaire scores were standardized on a scale from 0 to 100.

### 3.4 Study Procedures and Data Collection

For data collection, the Principal Investigator (PI), after telephone contact with the Top Manager of the participating centres, was personally responsible for participants' recruitment and information about the purpose of the study. The researcher asked participants to complete and return the questionnaires in paper format, allowing approximately 20 min for completion. To guarantee the confidentiality and anonymity, participants resubmitted the questionnaire in an envelope inside a special urn.

### 3.5 Statistical Analysis

To estimate the proportion of NMs' intention to leave, a sample size of at least 381 subjects was calculated using the method of confidence interval for a proportion with a precision level of ±5%, an expected value of 50%, on a population of approximately 39,000 NMs (FNOPI 2021).

The proportion of NMs' intention to leave was estimated by means of 95% Confidence Interval (95% CI) using binomial distribution.

A descriptive analysis was conducted to examine the characteristics of the subjects, their job and the items of the COPSOQII–short questionnaire. Subject characteristics included demographic information such as age, gender, marital status, number of children, and years of experience. Job characteristics regarded the work settings, current role, number of resources managed, region of working, total years worked in the current role. Mean and standard deviation or median and interquartile range (IQR) summarized the quantitative variables according to their distribution; absolute frequencies and proportions summarized the qualitative variables. The answer to the specific item of the questionnaire related to the intention to leave (How likely would you be to leave your current employment at your hospital within the next year?), was the outcome of interest and classified in two levels: (1) Unlikely (combining Unlikely and Not at all likely), (2) Likely (combining Extremely likely and Likely). Comparisons of subjects', work characteristics and questionnaire domains between “Likely” and “Unlikely” groups were conducted by using the Student' *t* or Wilcoxon sum‐rank test for continuous variables, and Chi‐square test for categorical variables.

To analyze the structure of the questionnaire, the internal consistency was assessed using Cronbach's alpha, considering a value of 0.70 or higher as good internal consistency. A confirmatory factor analysis (CFA) was also performed based on a four‐factor model (Relationship with management, Job demands, Interface with health, Supervisor support, Job control), as previously indicated (Setti et al. 2017b). The factor scores, calculated by the Bartlett method, were linearly related to the domain scores, thereby reducing the number of variables measured by the questionnaire. Goodness‐of‐fit indices, including the Comparative Fit Index (CFI), Tucker‐Lewis Index (TLI), and Root Mean Square Error of Approximation (RMSEA), were estimated to evaluate the model fit. CFI and TLI values closer to 0.95 and RMSEA values below 0.06 were considered indicative of a good model fit.

Latent Class Analysis (LCA) was conducted to identify subgroups (latent classes) within the study population, based on their responses to qualitative variables, specifically the intention to leave their job. The model assumes the existence of k latent classes, with each individual having a probability of belonging to each class. Conditional probabilities for the observed response patterns were estimated for each class. The number of latent classes was determined using the Bayesian Information Criterion. The Expectation–Maximization algorithm was used to estimate model parameters, and posterior probabilities were calculated to classify individuals into latent classes. This approach allowed for capturing unobserved heterogeneity in the population.

The factor scores were used in a logistic regression model to explore their association with the intention to leave. The outcome was the dependent variable, and the independent variables were the demographic and work characteristics of the subjects as well as the factor scores from the questionnaire. The number of resources managed and the number of years in the current role were dichotomized at 35 people and 10 years respectively.

### 3.6 Ethical Considerations

The study protocol was approved by the Ethics Committee (approval number: 2023 372). and by the Nursing Department of each participating hospital. All subjects were enrolled after providing informed consent. All data has been anonymized and stored securely.

The procedures for data collection and analysis were designed to ensure data confidentiality and in compliance with national and European regulations, including the Personal Data Act (Garante per la protezione dei dati personali 2013).

Administrative authorizations were obtained from all participating centres. Eligible participants received detailed information about the study's objectives, procedures, and data management prior to enrolment, and were interviewed only after providing consent. Additional information regarding ethical issues and the researcher's contact details was also provided by phone. The electronic data were stored in an access‐restricted folder, available only by the principal investigator.

## 4 Results

### 4.1 COPSOQ II—Short Evaluation

Confirmatory factor analysis showed a high level of structural integrity of the Italian version of COPSOQ‐II short form (Table S2) in four distinct factors. Table S4 reported the factor loading extracted from the CFA analysis. The first factor, “Relation with management”, was composed of Predictability, Rewards, Role clarity, Trust and Justice scales; factor 2, “Job Demands and Health Interface”, included Work Pace, Emotional demands, Work–family conflict, Self‐related health and Burnout scales; factor 3, “Supervisor support”, comprised two scales, Quality of leadership and Supervisor's support; factor 4, “Job Control”, was composed of Decision authority and Skill direction. Factors loadings related to Quantitative demand and Job satisfaction scales resulted below 0.3, hence not entering into the factor score estimation. Internal consistency for all four factors was satisfactory, with Cronbach's alpha values exceeding 0.5 (Table S3). The total scale Cronbach’s alpha was 0.88 (95% CI 0.87–0.89).

### 4.2 Study Results

Data were collected from 19 hospitals across 21 Regions. A total of 464 NMs were analyzed, 340 (73.3%) of which were females, with a median age of 52 years (IQR 47–57); most participants (68%) were married, with a median of 1 child (IQR 0–2), 40.7% with a master's degree or PhD. 284 NMs out of 464 (61%, 95% CI 57%–66%) declared their intention to leave.

Main subjects' and works' characteristics according to the probability of intention to leave are reported in Table 1. No differences were found between participants likely to leave the workplace and those unlikely to leave in terms of major demographic and work‐related characteristics. However, differences were evident in nearly all COPSOQ II—short form scales except Quantitative demand scale (Table 2).

**TABLE 1:** Subject characteristics according to the intention to leave.

Variables	All *n* = 464	Unlikely *n* = 180	Likely *n* = 284	*p*
Age, years (median, IQR)	52 (47; 57)	53 (49; 57)	52 (46; 57)	0.110^a^
Gender, F (*n*, %)	340 (73.3)	137 (76.1)	203 (71.5)	0.351^b^
Number of children (median, IQR)	1 (0; 2)	1 (0; 2)	2 (1; 2)	0.093^a^
Marital status (*n*, %)				0.500^b^
Married	314 (68.0)	118 (34.4)	196 (31.0)	
Divorced/single/widower	148 (32.0)	62 (65.6)	88 (69.0)	
Educational level (*n*, %)				0.145^b^
Undergraduate/other	168 (36.2)	75 (41.7)	93 (32.8)	
Bachelor	107 (23.1)	39 (21.7)	68 (23.9)	
Master degree/PhD	189 (40.7)	66 (36.7)	123 (43.3)	
Total years of working experience, (median, IQR)	30 (23; 36)	30 (22; 36)	30 (24; 36)	0.473^a^
Current work position (*n*, %)	*n* = 462	*n* = 179	*n* = 283	0.455^b^
Top manager	39 (8.4)	14 (7.8)	25 (8.8)	
Lower middle manager	340 (73.6)	130 (72.6)	215 (78.4)	
Upper middle manager	83 (18.0)	40 (22.3)	43 (15.2)	
Total years of work in current position (median, IQR)	6 (2; 14)	7 (2; 14)	6 (2; 13)	0.115^a^
Work setting (*n*, %)				0.485^b^
Medical/pediatric area	146 (31.5)	53 (29.4)	93 (32.8)	
Surgical/critical area	163 (35.1)	61 (33.9)	102 (35.9)	
Outpatients	155 (33.4)	66 (36.7)	89 (31.3)	
Geographic area (*n*, %)				0.272^b^
North	224 (48.3)	72 (40.0)	135 (47.5)	
Center	188 (40.5)	87 (48.3)	118 (41.6)	
South	52 (11.2)	21 (11.7)	31 (10.9)	
Number of workers managed (median, IQR)	35 (21; 65)	37 (25; 61)	30 (18; 84)	0.163^a^

*Note:* *p*: *p*‐value related to: ^a^Wilcoxon sum‐rank test, ^b^Chi‐square test.

Abbreviations: IQR, Interquartile range; Likely, Intending to leave; Unlikely, Not intending to leave.

**TABLE 2:** Questionnaire scores according to intention to leave.

COPSOQ II—short scales	All *n* = 464	Unlikely *n* = 180	Likely *n* = 284	*p*
Quantitative demand, score [median (IQR)] *Do you get behind with your work?* *Do you have enough time for your work tasks?*	50 (37.5; 50)	50 (37.5; 62.5)	50 (37.5; 50)	0.319
Work pace, score [median (IQR)] *Is it necessary to keep working at a high pace?* *Do you work at a high pace throughout all day?*	75 (75; 87.5)	75 (62.5; 87.5)	87.5 (75; 87.5)	0.005
Emotional demands, score [median (IQR)] *Do you get emotionally involved in your work?* *Do you have to relate to other people's personal problems as part of your work?*	75 (62.5; 87.5)	75 (62.5; 87.5)	75 (62.5; 87.5)	0.001
Decision authority, score [median (IQR)] *Do you have a large degree of influence on the decisions concerning your work?* *Can you influence the amount of work assigned to you?*	62.5 (50; 75)	62.5 (50; 75)	62.5 (37.5; 75)	0.002
Skill discretion, score [median (IQR)] *Do you have the possibility of learning new things through your work?* *Does your work require you to take the* initiative?	75 (62.5; 87.5)	75 (62.5; 87.5)	75 (62.5; 87.5)	0.011
Predictability, score [median (IQR)] *At your place of work, are you informed well in advance concerning for example important decisions, changes, or plans for the* future? *Do you receive all the information you need in order to do your work well?*	50 (37.5; 75)	62.5 (50; 75)	50 (37.5; 75)	< 0.001
Reward, score [median (IQR)] *Is your work recognized and appreciated by the management?* *Are you treated fairly at your workplace?*	50 (37.5; 62.5)	50 (46.8; 62.5)	37.5 (25; 62.5)	< 0.001
Role clarity, score [median (IQR)] *Does your work have clear objectives?* *Do you know exactly what is expected of you at work?*	50 (50; 75)	62.5 (50; 75)	50 (37.5; 62.5)	< 0.001
Quality of leadership, score [median (IQR)] *To what extent would you say that your immediate superior makes sure that the members of staff have good development opportunities?* *To what extent would you say that your immediate superior is good at work planning?*	50 (37.5; 75)	62.5 (50; 75)	50 (25: 65.6)	< 0.001
Supervisor support, score [median (IQR)] *How often is your nearest superior willing to listen to your problems at work, if needed?* *How often do you get help and support from your nearest superior, if needed?*	62.5 (37.5; 75)	75 (50; 87.5)	50 (37.5; 75)	< 0.001
Job satisfaction, score [median (IQR)] *How pleased are you with your job as a whole, everything is taken into consideration?*	66.7 (33.3; 66.7)	66.7 (66.7; 100)	66.7 (33.3; 66.7)	< 0.001
Work–family conflict, score [median (IQR)] *Do you feel that your work drains so much of my energy that it has a negative effect on your private life?* *Do you feel that your work takes so much of your time that it has a negative effect on your private life?*	66.7 (33.3; 83.3)	50 (33.3; 66.7)	66.7 (50; 83.3)	< 0.001
Trust, score [median (IQR)] *Can you trust the information that comes from the management?* *Does the management trust the employees to their work well?*	50 (37.5; 62.5)	50 (50; 62.5)	50 (25; 50)	< 0.001
Justice, score [median (IQR)] *Are conflicts resolved in a fair way?* *Is the work distributed fairly?*	50 (37.5; 62.5)	62.5 (50; 75)	50 (37.5; 62.5)	< 0.001
Self‐rated health, score [median (IQR)] *How is your general health?*	50 (50; 75)	75 (50; 75)	50 (50; 75)	< 0.001
Burnout, score [median (IQR)] *How often have you felt worn out?* *How often have you been emotionally exhausted?*	62.5 (50; 75)	50 (37.5; 75)	62.5 (50; 75)	< 0.001

*Note:* *p*: *p*‐value referred to Wilcoxon sum‐rank test.

Abbreviations: IQR, interquartile range; Likely, Intending to leave; Unlikely, Not intending to leave.

**FIGURE 1:**
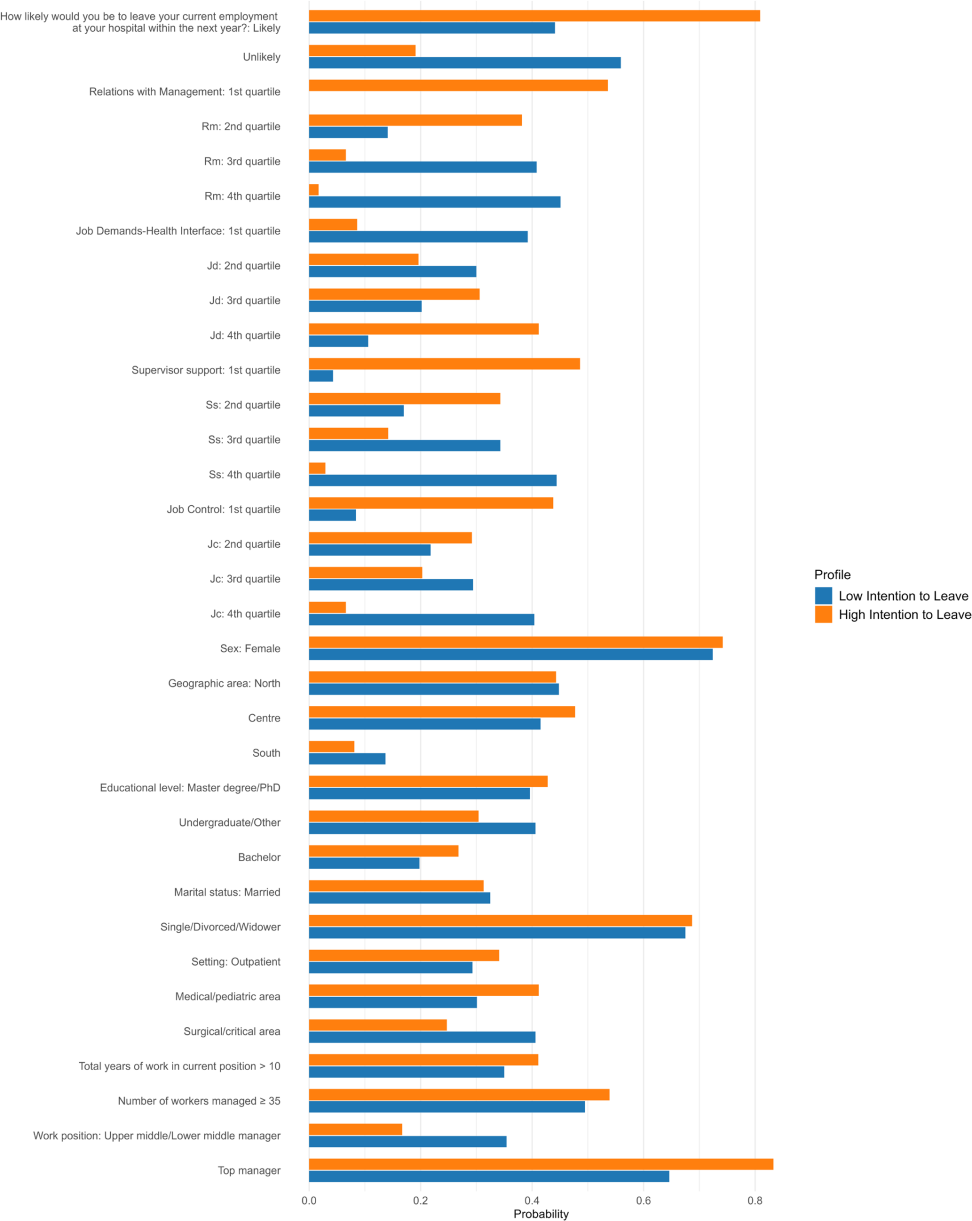
Profiles of nursing managers' intention to leave. Results of latent class analysis.

LCA identified two distinct latent classes among the participants, representing differing probabilities of leaving their current job (Figure 1). The analysis delineated two profiles:

1. First Profile (Low Intention to Leave): representing 54% of the sample, individuals in this class exhibited a low likelihood of intending to leave their position. They were predominantly employed in outpatient settings. These individuals demonstrated strong relationships with management, reported a good work‐life balance, and received significant support from supervisors. They also enjoyed high levels of job autonomy, all of which contributed to their lower intention to leave. This group had a 55.9% probability of being classified as “Unlikely” to leave, aligning with their more favorable working conditions and supportive environments.
2. Second Profile (High Intention to Leave): In contrast, the second profile, comprising 46% of the sample, included individuals with a high intention to leave their job. These individuals were more often employed in critical or surgical settings and reported poor relationships with management, low work‐life balance, and insufficient support from supervisors. Additionally, they reported low job autonomy, further contributing to their dissatisfaction. This group had an 80.9% probability of being classified as “Likely” to leave, reflecting their unfavorable work conditions and increased risk of turnover.

Table 3 presents the factor associated with the intention to leave (Likely vs. Unlikely). Higher scores in “Relations with Management” (factor 1) were associated with a 40% lower probability of leaving the workplace, whereas a higher score in Job Demands–Health Interface (Factor 2) was associated with a 56% higher probability of leaving. Subjects working in Northern regions reported a 58% higher probability of leaving the workplace compared to those working in a Central region. Educational level, work setting, current work position, other work characteristics, and individual socio‐demographic factors were not statistically associated with the intention to leave.

**TABLE 3:** Factors associated to intention to leave (yes vs. no): Results from the logistic regression.

Variables	OR	95% CI	*p*
Factor 1: Relations with management	**0.60**	**0.44; 0.809**	**0.001**
Factor 2: Job demands–health interface	**1.56**	**1.25; 1.956**	**< 0.001**
Factor 3: Supervisor support	0.90	0.68; 1.191	0.461
Factor 4: Job control	1.16	0.94; 1.438	0.173
Work setting			
Surgical/critical area vs. medical area	0.83	0.5; 1.387	0.478
Outpatients vs. medical area	0.90	0.54; 1.516	0.700
Current work position: Top manager vs. upper middle/lower middle manager	1.05	0.64; 1.718	0.854
Gender (male vs. female)	1.57	0.96; 2.62	0.076
Marital status: Single/divorced/widower vs. married	1.30	0.84; 2.03	0.240
Educational level: Undergraduate vs. master degree	0.71	0.44; 1.162	0.176
Bachelor vs. master degree	0.75	0.43; 1.325	0.322
Geographic area: Nord vs. centre	**1.58**	**1.01; 2.507**	**0.049**
South vs. centre	1.40	0.69; 2.896	0.351
Number of resources managed: ≥ 35 vs. < 35	1.27	0.81; 1.974	0.298
Total years in actual role: ≥ 10 vs. < 10	0.93	0.61; 1.412	0.730

Abbreviations: 95% CI, 95% Confidence interval; OR, Odd ratio. Bold values indicates statistical significance (*p* < 0.05).

## 5 Discussion

The main objective of this study is to estimate the prevalence of NMs who are willing to leave their jobs and identify associated factors such as subjects' socio‐demographic variables, workplace characteristics, and psychosocial work environment.

This study highlights the complexity of this phenomenon and the interplay between individual and organizational determinants. 284 nursing managers, out of 464 enrolled in the study, declared their intention to leave (61%, 95% CI 57%–66%), independently from subjects' socio‐demographic characteristics, work setting and current role. Our results are consistent with those of Warden et al. (2021) as they similarly found that intention to leave was similar among nurse managers (64.1%) nurse directors (64.9%) and nurse executives (66.6%). Differently, Hewko et al. (2015) and Lin et al. (2019) reported that, the intention of NMs to stay significantly exceeded their intention to leave, respectively of 89.1% and 70.5%. They reinforce the idea that work environment satisfaction is a valid predictor on intention to stay and that the principal factors reported by NMs intending to stay were work–life balance, sufficient support from their immediate supervisor and the ability to ensure quality of care. On the other hand, the critical factors for NMs intending to leave were work overload/work–life balance, insufficient ability to ensure the quality of patient care, lacking human/physical resources and inadequate empowerment to do their job.

Findings align with prior literature that identifies job dissatisfaction (Warshawsky and Havens 2014), emotional exhaustion (Warshawsky and Havens 2014), and workload (Mackoff 2011; Parsons and Stonestreet 2003; Cho et al. 2022) as factors influencing NMs' intentions to leave. Similarly, the studies of Catania et al. (2024) and Sasso et al. (2019) demonstrated that high workloads and emotional exhaustion directly link to the intention to leave for nurses.

As stated by Labrague (2024), in order to support NMs and mitigate turnover intentions, healthcare organizations need to prioritize the creation of a work environment that promotes work‐life balance and reduces psychological distress. According to Melnyk et al. (2025) feelings of burnout and mattering are closely linked perceived workplace wellness support, highlighting the importance of building a strong workplace wellness cultures and support for healthcare professionals. When nurse managers perceived higher culture of wellness and a greater sense of mattering, fewer reports of burnout and stress were observed (Melnyk et al. 2024). Consistent with this perspective, when nurse managers do not feel that they matter to their organization, they may become disengaged, which can ultimately increase their intention to leave (Melnyk et al. 2025). Our study reinforces the importance of positive organizational factors, such as managerial support and job autonomy, in mitigating turnover intentions. These findings are consistent with the RN4CAST project, where Aiken et al. (2011) observed that positive nurse‐physician relationships and strong organizational leadership were critical “pull” factors encouraging nurses to maintain their roles.

International literature further underscores the role of burnout as a significant driver of turnover intentions. McHugh et al. (2011) reported that emotional exhaustion correlates directly with higher turnover rates, advocating for interventions that target mental health and stress management among nursing staff. Barrientos‐Trigo et al. (2018) also confirmed the association of burnout with the likelihood of leaving, particularly when managerial support is lacking. These results are consistent with our findings on the need for supportive environments to mitigate the risk of burnout among nursing managers, especially those in high‐stress or critical care settings.

### 5.1 Strength and Limitation of the Study

Among other few international studies focusing on the intention of NMs to leave their jobs, our study has one of the highest numbers of participants, even though conducted at national level, ensuring a high level of accuracy of the estimated percentage of intentions to leave by NMs. Moreover, we took under consideration different work contexts, both medical, surgical, and critical care areas; additionally, pediatric and outpatient care settings were also included. Psychosocial work environment was assessed through the validated version of a questionnaire widely used in the scientific literature.

Among limitations, the cross‐sectional design limits the assessment of causal associations. A selection bias may be present if the most motivated nurse managers are those most likely to leave. Furthermore, the study relies on participants' self‐report questionnaire, thus the measures could be affected by a social desirability bias as subjects may select more socially acceptable answers rather than being truthful, or they may lack the means to properly self‐assess. In addition, although the overall internal consistency of the COPSOQ II‐short was high, some extracted factors showed only modest reliability, with Cronbach’s alpha values slightly above 0.5; therefore, findings related to these dimensions should be interpreted with caution.

### 5.2 Implication for Practice and Research

The COPSOQ‐II questionnaire, widely used in healthcare research, provides a comprehensive evaluation of work‐related psychosocial factors such as job demands, decision authority, and supervisor support. The periodic use of the COPSOQ‐II, as seen in studies by Siegrist and Marmot (2004), enables healthcare organizations to monitor shifts in the psychosocial environment over time. Such monitoring may facilitate the early identification of stressors and the timely implementation of targeted interventions. Moreover, qualitatively assessing the causes for NMs to leave their roles facilitates the identification of the areas in need of intervention. Undeniably, understanding the factors that influence turnover of nurse managers is pivotal to create and implement both strategy and intervention design to ensure sustainability of NMs.

### 5.3 Linking Evidence to Action

- Leaders should strengthen relations with management, including role clarity, recognition, trust, and organizational justice to support nurse manager retention.
- Healthcare organizations should address high job demands and work–health/work–family conflict, which were associated with higher intention to leave.
- Additional organizational support should be provided to nurse managers working in high‐intensity clinical areas, such as surgical and critical care settings.
- Routine assessment of the psychosocial work environment should be implemented to identify modifiable risk factors for intention to leave.

## 6 Conclusions

In conclusion, this study emphasizes the critical need for healthcare organizations to foster supportive work environments, to consider managerial well‐being as an essential component of workforce sustainability, and to address both personal and organizational factors influencing turnover intentions. Effective retention strategies should focus on strengthening relationships with management, promoting job autonomy, and ensuring manageable workloads. The regular assessment of the psychosocial environment using tools like the COPSOQ‐II is also requisite for supporting nursing managers' well‐being. These measures are essential both to retain skilled nursing managers and to maintain high standards of patient care.

## Conflicts of Interest

The authors declare no conflicts of interest.

## Supporting information


**Table S1:** Involved Italian hospitals.
**Table S2:** Confirmatory factor analysis.
**Table S3:** Factors loading from a confirmatory factor analysis (loading factors < 0.3 not shown).
**Table S4:** Internal consistency.

## Data Availability

The data that support the findings of this study are available from the corresponding author upon reasonable request.
