# Supplementary material for: Factors Influencing Intention to Leave Among Nurse Managers: A Cross‐Sectional Study
Source: Worldviews Evid Based Nurs. 2026 Apr 1;23(2):e70135. doi: 10.1111/wvn.70135 (PMC13044383; doi:10.1111/wvn.70135)
Supplement: Supplementary file 1 — Table S1: Involved Italian hospitals. Table S2: Confirmatory factor analysis. Table S3: Factors loading from a confirmatory factor analysis (loading factors < 0.3 not shown). Table S4: Internal consistency. [file WVN-23-0-s001.docx]

Supplementary material

| **Supplementary Table S1. Involved Italian Hospitals**   \| **Hospital** \| \| **Region** \| \| --- \| --- \| --- \| \|  \| \| Azienda ospedale Università Padova \| \| --- \| \| ASST Ospedale Papa Giovanni XXIII (Bergamo) \| \| Policlinico San Martino di Genova \| \| ASST SANTI PAOLO E CARLO (Bergamo) \| \| ASST Bergamo Ovest \| \| Azienda ospedaliera universitaria Meyer - IRCCS \| \| [ASST degli Spedali civili di Brescia (Brescia)](https://www.asst-spedalicivili.it/) \| \| Policlinico S.Orsola-Malpighi (Bologna) \| \| Fondazione Poliambulanza Istituto Ospedaliero (Bergamo) \| \| Azienda Ospedaliera San Camillo Forlanini (Roma) \| \| Istituto Giannina Gaslini (Genova) \| \| Ospedale Carlo Urbani (Jesi) \| \| ARNAS Garibaldi di Catania (Catania) \| \| Ospedale di Macerata (Macerata) \| \| Presidio ospedaliero San Salvatore di Pesaro (Pesaro) \| \| AOU delle Marche (Ancona) \| \| INRCA Ancona \| \| ASST Grande Ospedale Metropolitano Niguarda (Milano) \| \| [IRCCS Ospedale Sacro Cuore Don Calabria (Verona)](https://www.sacrocuore.it/) \| \| \| VENETO \| \| --- \| \| LOMBARDIA \| \| LIGURIA \| \| LOMBARDIA \| \| LOMBARDIA \| \| TOSCANA \| \| LOMBARDIA \| \| EMILIA-ROMAGNA \| \| LOMBARDIA \| \| LAZIO \| \| LIGURIA \| \| MARCHE \| \| SICILIA \| \| MARCHE \| \| MARCHE \| \| MARCHE \| \| MARCHE \| \| LOMBARDIA \| \| VENETO \| \|   **Table S2. Confirmatory factor analysis** | |
| --- | --- | --- | --- | --- | --- | --- | --- | --- | --- | --- | --- | --- | --- | --- | --- | --- | --- | --- | --- | --- | --- | --- | --- | --- | --- | --- | --- | --- | --- | --- | --- | --- | --- | --- | --- | --- | --- | --- | --- | --- | --- | --- | --- | --- | --- |
| *CFI* | 0.958 |
| *TLI* | 0.946 |
| *MSEA* | 0.06 (IC 95% 0.05-0.07) |
| **CFI**: Comparative Fix Index |  |
| **LTI**: Tucker Lewis Index |  |
| **RMSEA**: Root Mean Square Error of Approximation | |

**Table S3: Factors loading from a Confirmatory Factor Analysis (loadings factors less than 0.3 not shown)**

| **COPSOQ II – short scales** | **Factor 1 Relations with Management** | **Factor 2 Job Demands-Health Interface** | **Factor 3 Supervisor support** | **Factor 4 Job Control** |
| --- | --- | --- | --- | --- |
| Quantitative demand |  |  |  |  |
| Work pace |  | 0.431 |  |  |
| Emotional demands |  | 0.322 |  |  |
| Decision authority |  |  |  | 0.303 |
| Skill discretion |  |  |  | 0.713 |
| Predictability | 0.453 |  |  |  |
| Rewards | 0.512 |  |  |  |
| Role clarity | 0.516 |  |  |  |
| Quality of leadership |  |  | 0.888 |  |
| Supervisor’s support |  |  | 0.905 |  |
| Job satisfaction |  |  |  |  |
| Work-family conflict |  | 0.826 |  |  |
| Trust | 0.917 |  |  |  |
| Justice | 0.663 |  |  |  |
| Self-rated health |  | -0.517 |  |  |
| Burnout |  | 0.783 |  |  |

**Table S4. Internal consistency**

| **COPSOQ II – short scales** | | **Median (IQR)** | **Cronbach's α (95%CI)** |
| --- | --- | --- | --- |
| ***Factor 1: Relations with Management*** | |  | 0.86 (0.84; 0.88) |
|  | Predictability, score [median (IQR)] | 50 (37.5; 55.2) | 0.84 (0.82; 0.86) |
|  | Rewards, score [median (IQR)] | 50 (37.5; 46.7) | 0.83 (0.80; 0.85) |
|  | Role Clarity, score [median (IQR)] | 50 (50; 57.2) | 0.85 (0.82; 0.87) |
|  | Trust, score [median (IQR)] | 50 (37.5; 48.1) | 0.82 (0.79; 0.85) |
|  | Justice, score [median (IQR)] | 50 (37.5; 52) | 0.84 (0.82; 0.86) |
| ***Factor 2: Job Demands-Health Interface*** | |  | 0.73 (0.69-0.77) |
|  | Work Pace, score [median (IQR)] | 75 (75; 78.7) | 0.73 (0.69; 0.77) |
|  | Emotional Demands, score [median (IQR)] | 75 (62.5; 75.2) | 0.73 (0.69; 0.77) |
|  | Work-Family Conflict, score [median (IQR)] | 66.7 (33.3; 60.4) | 0.62 (0.56; 0.67) |
|  | Self-rated health, score [median (IQR)] | 50 (50; 59.8) | 0.69 (0.65; 0.74) |
|  | Burnout, score [median (IQR)] | 62.5 (50; 58.5) | 0.61 (0.55; 0.66) |
| ***Factor 3: Supervisor support*** | |  | 0.89 (0.86; 0.90) |
|  | Quality of leadership, score [median (IQR)] | 50 (37.5; 53.5) |  |
|  | Supervisor support, score [median (IQR)] | 62.5 (37.5; 60.4) |  |
| ***Factor 4: Job Control*** | |  | 0.54 (0.44; 0.61) |
|  | Decision authority, score [median (IQR)] | 62.5 (50; 58.9) |  |
|  | Skill discretion, score [median (IQR)] | 75 (62.5; 74.4) |  |

IQR: Interquartile Range
